# Supplementary material for: The genome sequence of the Common Pochard, Aythya ferina (Linnaeus, 1758)
Source: Wellcome Open Res. 2025 Mar 19;10:152. [Version 1] doi: 10.12688/wellcomeopenres.23904.1 (PMC12009480; doi:10.12688/wellcomeopenres.23904.1)
Supplement: Peer review history

# Reviewer report: Reviewer response for version 1

**DOI:** 10.21956/wellcomeopenres.26372.r121114

**Published:** 2025-04-19

**Authors:** Kritika M Garg

**Affiliations:** 1 Ashoka University, Sonipat, India

The authors describe the genome of common pochard, a vulnerable species important for the spread of highly pathogenic avian influenza. The genome sequencing is methods is well described. As RNA sequencing was carried out, it would be important to also perform the annotation and describe it along with the genome.

Are sufficient details of methods and materials provided to allow replication by others?

Yes

Is the rationale for creating the dataset(s) clearly described?

Yes

Are the datasets clearly presented in a useable and accessible format?

Yes

Are the protocols appropriate and is the work technically sound?

Yes

Reviewer Expertise:

Population genomics and comparative genomics

I confirm that I have read this submission and believe that I have an appropriate level of expertise to confirm that it is of an acceptable scientific standard.

# Reviewer report: Reviewer response for version 1

**DOI:** 10.21956/wellcomeopenres.26372.r121106

**Published:** 2025-04-10

**Authors:** Leif Andersson

**Affiliations:** 1 Medical Biochemistry and Microbiology, Uppsala University, Uppsala, Sweden

The paper provides a high quality, chromosome level assembly of the Common Pochard. A valuable resource for research on this species. I have only minor comments to make.

 1. I think it would be be useful if the authors make a comparison with the recently published chromosome level assembly for this species published by Xia et al. (2024). It would be good to compare quality, contiguity of the two assemblies and identification of sex chromosomes.

 2. End of Background. I suggest to change the following text ” that are likely to be associated with diving adaptations” to ” that may be associated with diving adaptations”, because it is difficult to infer evolutionary processes from a genome sequence.

Are sufficient details of methods and materials provided to allow replication by others?

Yes

Is the rationale for creating the dataset(s) clearly described?

Yes

Are the datasets clearly presented in a useable and accessible format?

Yes

Are the protocols appropriate and is the work technically sound?

Yes

Reviewer Expertise:

Evolutionary genomics

I confirm that I have read this submission and believe that I have an appropriate level of expertise to confirm that it is of an acceptable scientific standard.

## References

[1] : Chromosome-level genome provides insights into evolution and diving adaptability in the vulnerable common pochard (Aythya ferina). *BMC Genomics* .2024;25(1) : 10.1186/s12864-024-10846-6 927 10.1186/s12864-024-10846-6 39363174 PMC11451245
